# Global Regulator IscR Positively Contributes to Antimonite Resistance and Oxidation in *Comamonas testosteroni* S44

**DOI:** 10.3389/fmolb.2015.00070

**Published:** 2015-12-18

**Authors:** Hongliang Liu, Weiping Zhuang, Shengzhe Zhang, Christopher Rensing, Jun Huang, Jie Li, Gejiao Wang

**Affiliations:** ^1^State Key Laboratory of Agricultural Microbiology, College of Life Science and Technology, Huazhong Agricultural UniversityWuhan, China; ^2^Shandong Provincial Research Center for Bioinformatic Engineering and Technique, School of Life Sciences, Shandong University of TechnologyZibo, China; ^3^Department of Plant and Environmental Sciences, University of CopenhagenFrederiksberg, Denmark

**Keywords:** IscR, *Comamonas testosteroni*, multi-metal resistance, antimonite oxidation, oxidative stress, iron-sulfur cluster

## Abstract

Antimonial compounds can be found as a toxic contaminant in the environment. Knowledge on mechanisms of microbial Sb oxidation and its role in microbial tolerance are limited. Previously, we found that *Comamonas testosteroni* S44 was resistant to multiple heavy metals and was able to oxidize the toxic antimonite [Sb(III)] to the much less toxic antimonate [Sb(V)]. In this study, transposon mutagenesis was performed in *C. testosteroni* S44 to isolate genes responsible for Sb(III) resistance and oxidation. An insertion mutation into *iscR*, which regulates genes involved in the biosynthesis of Fe-S clusters, generated a strain called iscR-280. This mutant strain was complemented with a plasmid carrying *iscR* to generate strain iscR-280C. Compared to the wild type S44 and iscR-280C, strain iscR-280 showed lower resistance to Sb(III) and a lower Sb(III) oxidation rate. Strain iscR-280 also showed lower resistance to As(III), Cd(II), Cu(II), and H_2_O_2_. In addition, intracellular γ-glutamylcysteine ligase (γ-GCL) activity and glutathione (GSH) content were decreased in the mutated strain iscR-280. Real-time RT-PCR and *lacZ* fusion expression assay indicated that transcription of *iscR* and *iscS* was induced by Sb(III). Results of electrophoretic mobility shift assay (EMSA) and bacterial one-hybrid (B1H) system demonstrated a positive interaction between IscR and its promoter region. The diverse defective phenotypes and various expression patterns suggest a role for IscR in contributing to multi-metal(loid)s resistance and Sb(III) oxidation via Fe-S cluster biogenesis and oxidative stress protection. Bacterial Sb(III) oxidation is a detoxification reaction.

## Introduction

Antimony (Sb) belongs to subgroup 15 of the periodic table along with nitrogen (N), phosphorus (P), arsenic (As), and bismuth (Bi). The most common oxidation states found in nature are Sb(III) and Sb(V) (Li et al., 2013). Sb and its compounds are considered as pollutants by the Environmental Protection Agency of the United States (USEPA) and the European Union (Herbst et al., 1985; Filella et al., 2002). Nowadays, Sb is dramatically increased in bogs and arctic polar ice cores showing the serious degree of Sb pollution due to anthropogenic activities (Shotyk et al., 2005). Molecular mechanisms of Sb resistance were mostly studied in the protozoan parasite *Leishmania*. *In vitro* studies suggested that genes encoding AQP1, PGPA, TDR1, and ACR2 in parasites are required for Sb(III) resistance (Decuypere et al., 2012). The best-known mechanism of resistance to Sb involves the detoxification of Sb(III) via conjugation to trypanothione [T(SH)2], which is a thiol compound in the protozoan parasite (Legare et al., 2001).

Sb shares similar chemical and toxicological properties with arsenic. However, unlike bacteria-arsenic interactions, bacteria-Sb interactions have not been elucidated in great detail. The glycerol transporter GlpF in *Escherichia coli* is reported to be responsible for Sb(III) uptake (Meng et al., 2004), while the ArsB protein, Acr3p family, and ABC transporter superfamily are involved in Sb(III) efflux (Filella et al., 2007). To our knowledge, only a few studies have reported biological Sb(III) oxidation, either as a cellular detoxification mechanism in bacteria and algae (Torma and Gabra, 1977; Lehr et al., 2007) or as a possible chemo-autotrophic process for the bacterium *Stibiobacter senarmontii* (Lialikova, 1974). Recently, the arsenite oxidase AioAB in *Agrobacterium tumefaciens* was found to be able to oxidize Sb(III) (Wang et al., 2015). The oxidoreductase AnoA was reported to responsible for bacterial Sb(III) oxidation (Li et al., 2015). However, the disruption of both of these genes only led a partial loss of Sb(III) oxidation, thereby indicating the existence of unknown mechanisms related to bacterial Sb(III) oxidation.

Three regulatory systems have been reported to affect Fe-S cluster assembly, Isc (iron-sulfur cluster), Suf (sulfur formation), and Nif (nitrogen fixation) (Tokumoto and Takahashi, 2001). Among them, the Isc system, encoded by the *isc* operon (*iscRSUA–hscBA–fdx*; *iscR*, [Fe-S] assembly transcription factor; *iscS*, cysteine desulfurase; *iscU*, [Fe-S] assembly scaffold; *iscA*, [Fe-S] assembly protein; *hscBA*, [Fe-S] protein assembly chaperones; and *fdx*, ferredoxin), has been most extensively studied in *E. coli* (Schwartz et al., 2001; Giel et al., 2013; Rajagopalan et al., 2013). In *E. coli*, IscR is a transcriptional repressor of *isc* operon exhibiting two major forms: apo-IscR lacks [2Fe-2S] and holo-IscR possesses [2Fe-2S] (Schwartz et al., 2001). Further analysis of IscR-regulated promoters in *E. coli* directly revealed two classes of IscR binding motifs, type I and type II (Giel et al., 2006). Holo-IscR binds to both types of motifs, whereas apo-IscR only binds to the type II motif (Rajagopalan et al., 2013).

IscR functions as a sensor of cellular Fe-S cluster levels under both stressful and physiological conditions, such as oxidative stress and iron availability (Giel et al., 2013; Romsang et al., 2014). Studies have shown that IscR regulates more than 40 genes including genes encoding additional proteins involved in Fe-S cluster biogenesis, anaerobic respiratory [Fe-S] enzymes, biofilm formation, and virulence (Giel et al., 2006; Lim and Choi, 2014). These findings indicated that IscR is a global regulator playing broader roles than only modulating Fe-S cluster assembly in bacteria. Nonetheless, the regulatory pattern of IscR in *Comamonas testosteroni* and IscR involvement in Sb(III) resistance/oxidation have not yet been characterized.

*C. testosteroni* S44 was isolated from soil of a Sb mine in Lengshuijiang City, central south China, where the soil was severely contaminated with multiple metal(loid)s (Xiong et al., 2011). Strain S44 has the ability to oxidize Sb(III) to Sb(V) and resist to multiple metal(loid)s (Li et al., 2013). Whole genome sequencing of *C. testosteroni* S44 was performed and showed multiple metal resistance genes to Sb, As, Cd, and Cu (Xiong et al., 2011). In this study, in order to analyze mechanism for Sb(III) resistance and oxidation, transposon mutagenesis was first performed based on Sb(III) sensitive phenotypes. An *iscR* mutant, designated strain iscR-280, exhibited decreased resistance to Sb(III), As(III), Cd(II), Cu(II), H_2_O_2_, and lower Sb(III) oxidation rate. Moreover, the γ-glutamylcysteine ligase (γ-GCL) activity and glutathione (GSH) content was reduced in strain iscR-280, which was suggested to be important in Sb(III) resistance. IscR exogenously provided on a multi-copy plasmid in strain iscR-280C nearly restored all the defective phenotypes. Results of real-time RT-PCR and *lacZ* fusions revealed the essential role of IscR by sensing the cellular [Fe-S] demands. Moreover, EMSA and B1H assay demonstrated DNA binding properties of IscR.

## Materials and methods

### Bacterial strains, transposon mutagenesis, and complementation

Bacterial strains and plasmids used in this study are listed in Table 1. Primers are available in Table S1 (see Supplementary Material). To create a random transposon mutant library of *C. testosteroni* S44, *E. coli* S17-1 λ*pir* strain (Simon et al., 1983) containing pRL27-Cm, a suicide vector possessing a hyper-transposable Tn5 element (Miller and Mekalanos, 1988), was used as a conjugal donor for strain S44. To obtain Sb(III) sensitive mutants, the colonies from the LB plates were inoculated onto a chemically defined medium A (CDM-A, Weeger et al., 1999) plate with 50 μg/mL chloramphenicol (Cm) + 50 μg/mL kanamycin (Km) + 100 μM Sb(III) [K_2_Sb_2_(C_4_H_4_O_7_)_2_]. The plates were incubated at 28°C for 4 d. The genomic DNA regions flanking the Tn5 insertion of the selected Sb(III) sensitive mutants were obtained by inverse PCR and confirmed by DNA sequencing. To demonstrate that the defective phenotype of the lower Sb(III) resistance is due to an *iscR* insertion, an *iscR* complementation was performed using the PCR amplified *iscR* coding sequence (Table S1) digested with *Kpn*I and *Xba*I and subcloned into a broad host-range vector pCPP30 (Huang et al., 1992) to form plasmid pCPP30::*iscR*. The pCPP30::*iscR* was transferred into strain iscR-280 by conjugation to yield the complemented strain iscR-280C.

**Table 1:** **Bacteria and plasmids used in this study**.

**Strains and plasmids**	**Relevant property or derivation**	**Source or reference**
*Comamonas testosteroni* S44	Wild type, Sb(III) oxidizing Rif^r^, Cm^s^, Tet^s^	This study
iscR-280	*iscR* mutant with Tn5 insertion Rif^r^, Cm^r^, Tet^s^	This study
iscR-280C	*iscR* complementary strain Rif^r^, Cm^r^, Tet^r^	This study
***Escherichia coli***		
DH5α (λ*pir*)	Φ80d*lacZ* ΔM15 Δ(*argF-lacZYA*) *U169 relA1 hsdR17 deoR thi-1 supE44 gyrA96 recA1/*λ*pir*	Miller and Mekalanos, 1988
S17-1 (λ*pir*)	*Tp^*r*^ Str^*r*^ recA thi pro hsdR^−^ hsdM^+^* RP4, 2Tc, Mu, Km, T7, λ*pir*	Simon et al., 1983
BL21(DE3)	F^−^*omp*T *hsd*S_B_(r${}_{\text{B}}^{-}$m${}_{\text{B}}^{-}$) *gal dcm* (DE3)	Laboratory collection
DH5α (pLSP-kt2lacZ) DH5α (PiscR′-lacZ) DH5α (PiscR′-lacZ-S44) DH5α (PiscR′-lacZ-280) XL1-Blue	DH5α with empty *LacZ*-fusion vector pLSP, Km^r^ DH5α containing pLSP with *iscR* promoter region, Km^r^ DH5α containing pLSP with *iscR* and *iscR* promoter region, Km^r^ DH5α containing pLSP with mutant *iscR* and *iscR* promoter region, Km^r^ B1H system reporter strain, Km^r^	This study This study This study This study Guo et al., 2009
CK+	Positive control of B1H system composed of co-transformants with pBX-Mt2031p/pTRG-Rv3133c, Km^r^, Cm^r^, Tet^r^, Str^r^	Guo et al., 2009
CK-	Negative controls of B1H system composed of co-transformants with pBXcmT/pTRG, pBX-*iscR*p/pTRG and pBXcmT/pTRG-IscR, Km^r^, Cm^r^, Tet^r^	This study
pTRG-IscR/pBXcmT-*iscR*p	XL1-Blue containing pTRG-*iscR* and pBXcmT with *iscR* promote region, Km^r^, Cm^r^, Tet^r^, Str^r^	This study
**PLASMIDS**		
pRL27-Cm	Transposon vector, *ori*R6K, Cm^r^	This study
pCPP30	Broad host complementary vector, *tetA*	Huang et al., 1992
pCPP30::*iscR* pLSP-kt2lacZ pTRG pBXcmT	pCPP30 with 680 bp long DNA sequence cloned from strain S44, starting from +81 bp relative to *iscR* start codon, Tet^r^ Km^r^ *oriV*, LacZ-fusion vector B1H system vector, *tetA* B1H system vector, Cm^r^	This study Kang et al., 2012 Guo et al., 2009 Guo et al., 2009
pET-28a(+) pET-28a(+)-iscR	Expression vector for IscR pET-28a(+) with *iscR* coding region	Novagen, Madison, WI This study

*Rif^r^, rifampicin resistant; Cm^r^, chloramphenicol resistant; Tet^r^, tetracycline resistant; Km^r^, kanamycin resistant; Tp^r^, trimethoprim resistant; Str^r^, streptomycin resistant; Cm^s^, chloramphenicol sensitive; Tet^s^, tetracycline sensitive*.

### Sb(III) oxidation test and resistance to metal(loid)s and H_2_O_2_

Sb(III) oxidation rates were tested using HPLC coupled with a hydride-generation atomic fluorescence spectroscopy (HPLC-HG-AFS, Beijing Titan Instruments Co., Ltd., China) as described previously (Lehr et al., 2007). For growing cells, overnight cultures of strains S44, iscR-280, and iscR-280C (OD_600_~1.0) were inoculated into CDM-A liquid medium supplemented with 50 μM Sb(III) and incubated at 37°C for 7 d. For culture supernatant and cell-free extract, overnight cultures (100 mL) was harvested (OD_600_~1.0) by centrifugation and two equal parts of culture supernatant and cell-free extract were supplemented with 1 μM Sb(III) and incubated at 37°C for 5 d. Culture supernatant and cell-free extract were prepared as described previously (Liu et al., 2015). Minimum inhibitory concentration (MIC) was performed in LB medium supplemented without Sb(III) and with different concentration of NaAsO_2_[As(III)], CuSO_4_[Cu(II)], and CdCl_2_ [Cd(II)] at 37°C for 48 h. Spotting assays using serially diluted bacterial suspensions were performed to determine the cellular resistance to H_2_O_2_ (0.3 mM).

### Co-transcription of *iscRSUA* and IscR-DNA interaction analysis

**Figure 1:**
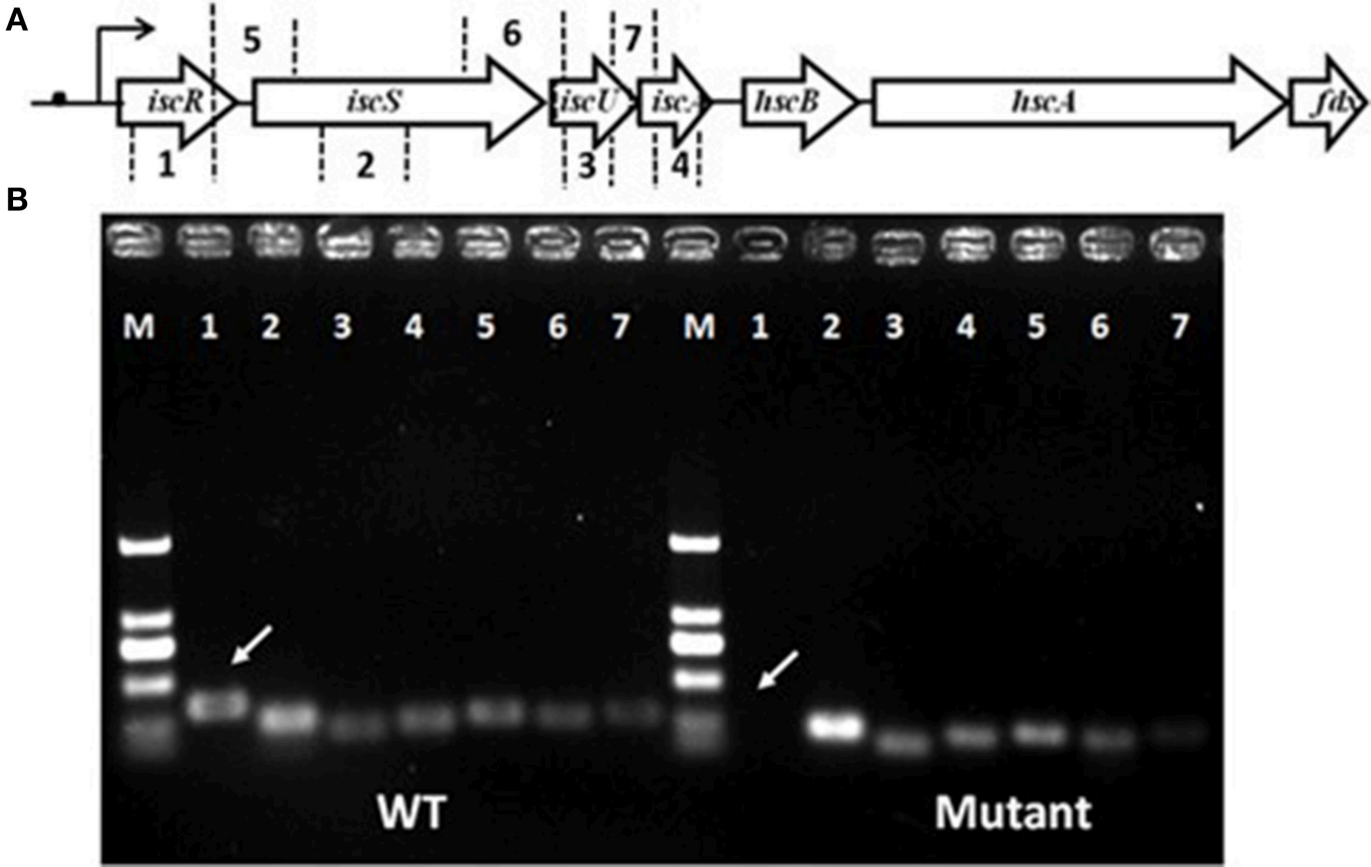
**Gene organization of the *isc* operon and schematic representation of *iscRSUA* co-transcription**. **(A)** The *isc* operon of strain S44 was composed of the *iscRSUA–hscBA–fdx* genes (located in contig61 with accession number, ADVQ01000059; protein_id, EFI60623–EFI60629). **(B)** DNA electrophoresis of *iscRSUA* for co-transcription assay of strains S44 (WT) and iscR-280 (mutant). The white arrows marked for cDNA of *iscR* in S44 and iscR-280. The hollow arrows represent predicted coding regions of the Isc cluster genes. No. 1–7 represent coding regions of *iscR* (348 bp), *iscS* (253 bp), *iscU* (204 bp), and *iscA* (242 bp), and cross-linked regions of *iscR*-*S* (270 bp), *iscS*-*U* (253 bp), and *iscU-A* (288 bp) selected for co-transcription analysis, respectively. The black dot, curved arrow, and M represent putative type I IscR-binding motif, transcription initiation and DNA marker, respectively.

Total RNA was isolated using Trizol reagent (Invitrogen, Grand Island, NY, USA) and purified by RNA clean-up kit (Omega Bio-Tek, Georgia, USA) according to the manufacturer's instructions. Confirmation of no genomic DNA contaminating the RNA was also performed. The ranges of coding sequences used for *in vitro* co-transcription of *iscRSUA* were designed as described in Figure 1.

The *iscR* promoter region was predicted using Berkeley Drosophila Genome Project (BDGP, http://www.fruitfly.org/seq_tools/promoter.html, Reese, 2001). The potential binding motifs of IscR in different species were further analyzed by comparing with a 25 bp consensus sequence extracted from the RegPrecise database (http://regprecise.lbl.gov/RegPrecise/sites.jsp?regulog_id=1935, Novichkov et al., 2010). Multiple alignments of IscRs from different bacteria were performed using Clustal_X algorithm (Thompson et al., 1997).

A B1H system was constructed to detect *in vivo* IscR-DNA interactions. A 200 bp PCR product of predicted *iscR* promoter region was subcloned into pBXcmT (Guo et al., 2009) to yield pBX-*iscR*p. A 600 bp PCR product of *iscR* coding region was subcloned into pTRG (Guo et al., 2009) to yield pTRG-IscR. Then, pBX-*iscR*p and pTRG-IscR were co-transformed into the *E*. *coli* XL1-Blue MRF' Kan reporter strain (XL1-Blue, Stratagene). Rv3133c has been previously shown to bind to motif sequences upstream of the *acr* (Rv2031 = Mt2031) coding region and its C-terminal HTH DNA-binding domain is essential for the binding. The sequence-specific interaction between Rv3133c and the promoter of Mt2031 was commonly used as a positive control to test the new B1H system (Guo et al., 2009; Gao et al., 2012). Co-transformants containing pBXcmT/pTRG-IscR, pBX-*iscR*p/pTRG and pBXcmT/pTRG were used as negative controls. The transformants were incubated on His-selective medium (Guo et al., 2009) that was composed of M9 minimal medium supplemented with 0.4% glucose, 200 μM adenine-HCl, 0.1% His Do sup [His Dropout Supplement (BD/Clontech, Cat. #630415)], 100 μM CaCl_2_, 400 μM IPTG, 1 mM MgSO_4_, 1 mM Thiamine HCl, 10 μM ZnSO_4_, 50 μg/ml Km, 50 μg/ml Cm, 12.5 μg/ml tetracycline (Tet), 5 mM 3-amino-1,2,4-triazole (3-AT) and 16 μg/ml streptomycin (Str). LB medium supplemented with 50 μg/ml Km, 50 μg/ml Cm, and 12.5 μg/ml Tet was used as a control. His-selective plate and LB agar plate were aerobically incubated at 28°C for 7 d.

EMSA was also performed to confirm the *in vitro* interaction between IscR and its promoter. The intact coding region of *iscR* was subcloned into a His-tag expression vector, pET-28a(+) (Novagen, Madison, WI), to yield pET-28a(+)-IscR (Table 1). The His-tagged IscR was then expressed in *E. coli* BL21 (DE3), and purified by affinity chromatography (Ni-Sepharose 6 Fast Flow, GE Healthcare, UK). Each DNA (0.2 μg) was incubated with the purified IscR (0.8–6.4 μg) for 30 min at 30°C in a 20 μL reaction mixture containing binding buffer [100 mM HEPES, pH 7.6, 5 mM EDTA, 50 mM (NH_4_)_2_SO_4_, 5 mM DTT, Tween 20, 1% (w/v), 150 mM KCl]. The binding reaction was stopped by the addition of 4 × loading buffer [0.25 × TBE buffer, 60%; glycerol, 40%, bromphenol blue, 0.2% (w/v)]. Predicted *iscR* promoter region coupled with heat inactivated IscR (3.2 μg) and the non-specific DNA sequence coupled with intact IscR were used as negative controls. Electrophoretic analysis of the IscR-DNA complexes were performed in 6% non-denatured PAGE stained with ethidium bromide. To test the exact binding sequence of IscR within the promoter region of *iscRSUA*, four 30-bp-DNA sequences with conserved and mutational motifs were performed with EMSA.

### Real-time RT-PCR analysis

Strains S44, iscR-280, and iscR-280C were inoculated into 100 mL liquid LB medium incubated at 37°C with shaking at 160 rpm for 9 h (exponential phase), then the inoculum was induced by addition of 50 μM of Sb(III) for 1 h. The bacterial culture without induction was used as a control. Total RNA was extracted and purified as described above. Amplification of cDNA was performed using PrimeScript RT-PCR Kit II (Takara BioInc, Shiga, Japan) with random primers following the manufacturer's instructions. Real-time RT-PCR was performed with SYBR in triplicate using Applied Biosystems® ViiA™ 7 Real-Time PCR System (Life Technologies, Carlsbad, CA, USA) with the specific primers listed in Table S1. Quantitation of transcripts of *iscR* was performed using ΔCt method (Pfaffl, 2001) with the 16S rRNA gene for normalization.

### Reporter gene fusions for IscR regulation assay

pLSP-kt2*lacZ* vector (Kang et al., 2012) was used to identify the IscR regulated transcription initiation of the *isc* operon. PCR fragments containing −402 to +128 nucleotides relative to the *iscR* start codon; −405 to +518 nucleotides relative to the *iscR* start codon; and −207 to +537 nucleotides relative to the *iscR* start codon, with −76 to +130 nucleotides relative to the *iscS* start codon of iscR-280, were ligated separately into pLSP-kt2*lacZ* to generate *lacZ* fusion plasmids. These *lacZ* fusion plasmids were referred to as PiscR′-*lacZ*, PiscR′-*lacZ*-S44, and PiscR′-*lacZ*-280, respectively. PiscR′-*lacZ* and PiscR′-*lacZ*-S44 contained the predicted intact *iscR* promoter region, while PiscR′-*lacZ*-280 contained an *iscR* with Tn5 insertion. The empty vector pLSP-kt2*lacZ* was used as a control. In each case, the resulting *lacZ* fusion was introduced into *E. coil* strain DH5α and bacterial cells were grown in LB medium for 10 h. β-galactosidase activity assays were performed using the method described previously (Miller, 1972).

### Measurement of γ-GCL activity and GSH content

Strains were incubated in LB medium at 37°C and harvested at incubation time of 10 h and 12 h. Cell pellets were lysed by a brief sonication and clarified by centrifugation as previously described (Liu et al., 2015). Soluble protein concentrations were determined by Bradford assay. Since γ-GCL is the rate-limiting enzyme for GSH synthesis, both GCL activity and GSH content of cellular extracts were determined as described previously (White et al., 2003). This method relies upon the reaction between γ-glutamylcysteine (γ-GC) or GSH with a highly specific fluorogenic probe, naphthalene-2, 3-dicarboxaldehyde (NDA). Briefly, the cellular extracts (50 μl) used for GCL activity and GSH content were incubated at 37°C in a microtiter plate with GCL reaction mixture. GCL reaction was initiated by 1 mM cysteine for 30 min. Reactions were halted and proteins were precipitated by adding 200 mM of 5-sulfosalicylic acid (SSA). Then cysteine was added into the microtiter plates for GSH content assay. Aliquots of each well were derivatized with NDA in the dark for 30 min at room temperature. The fluorescence (472 nm ex/528 nm em) was measured on an EnVision® Multilabel Reader (Perkinelme, Waltham, MA, USA).

## Results

### Isolation and identification of Sb(III) sensitive mutants

Transposon mutagenesis was performed using a Tn5-tagging method as described previously (Larsen et al., 2002). Approximately 5000 transformants were isolated and tested for Sb(III) resistance on CDM-A agar plates. Six mutants with different Tn5 insertion position within *iscRS* were found. Four mutants have an insertion within *iscR* and two mutant have an insertion between *iscR* and *iscS*. All of the six mutants showed a lower resistance to Sb(III) (data not shown). A mutant strain iscR-280 showed the lowest Sb(III) resistance and was chosen for this study.

Analysis of the *C. testosteroni* S44 genome (ADVQ00000000, Xiong et al., 2011) revealed only one Isc system containing *iscRSUA-hscBA*-*fdx* (protein_id: EFI60623–EFI60629, ADVQ01000059, Figure 1A) putatively involved in Fe-S cluster biogenesis. *In vitro* transcription assays indicated that *iscR* was co-transcribed with *iscSUA* (Figure 1B). No genomic DNA contaminated the total RNA (Figure S1). Multiple alignments of bacterial IscRs (Figure S2) exhibited an analogous organization among different species, such as *E. coli, V. vulnificus, A. vinelandii, E. chrysanthemi*, and *P. aeruginosa* (Rincon-Enriquez et al., 2008; Lim and Choi, 2014; Romsang et al., 2014). The residues (C92, C98, C104, and H107) involved in [Fe-S] ligation (Fleischhacker et al., 2012) were all highly conserved.

### Defective phenotypes were restored by *iscR*-complementation

**Figure 2:**
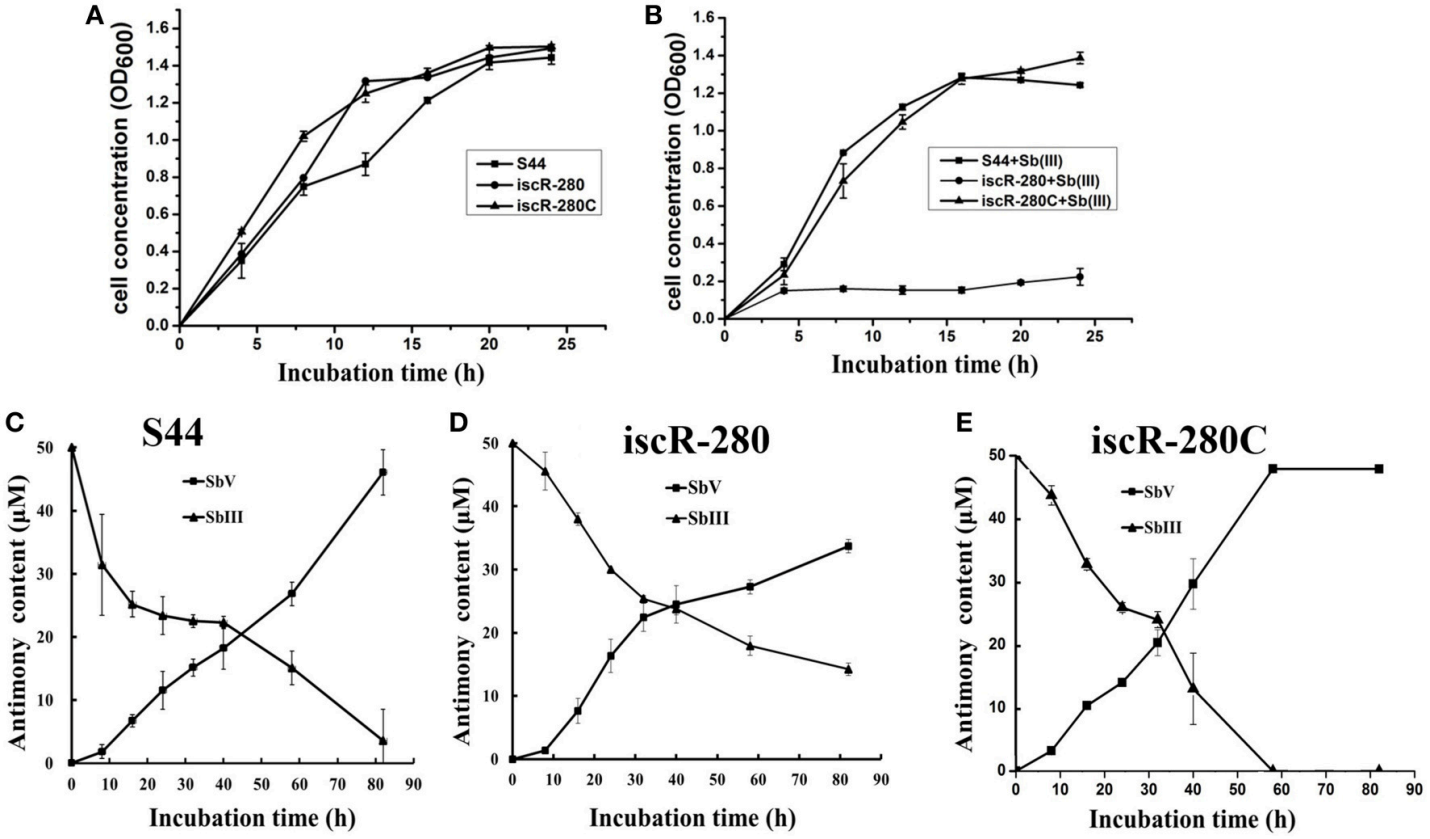
**Cell growth and Sb(III) oxidation assays. (A)** and **(B)** represent growth curves of S44 (■), iscR-280(∙), and iscR-280C (▴) incubated in LB medium without Sb(III) or supplemented with 100 μM Sb(III), respectively. **(C,D)**, and **(E)** represent Sb(III) oxidation curves of strains S44, iscR-280, and iscR-280C, respectively. Content of Sb(III) (▴) and Sb(V) (■) in **(C,D)**, and **(E)** was measured simultaneously using HPLC-HG-AFS. Data are expressed as mean ± SD, *N* = 3. Error bars represent standard deviations of triplicate tests.

An *iscR* complemented strain iscR-280C was generated as described above. Growth, multi-metal(loid)s resistance and Sb(III) oxidation rates were examined in strains S44, iscR-280, and iscR-280C. Without addition of Sb(III), strain iscR-280 showed the same growth rate in LB medium compared with strains S44 and iscR-280C (Figure 2A). However, growth of strain iscR-280 was strongly inhibited when Sb(III) was added (Figure 2B). Assays of bacterial Sb(III) oxidation rates were performed using liquid CDM-A which by itself exhibited no Sb(III) oxidation. The Sb(III) oxidation rate of strain iscR-280 in CDM-A medium was much lower than strains S44 and iscR-280C (Figures 2C–E), which might at least in part be attributed to diminished growth of strain iscR-280. Furthermore, Sb(III) oxidation occurred in the culture supernatant with the oxidation rates being highest in S44 > iscR-280C > iscR-280 (Figure S3A). The cell-free extract as a control showed no significant Sb(III) oxidation (Figure S3B), which suggested Sb(III) oxidation mainly occurred outside of cells. Strain iscR-280 displayed lower MICs compared to strain S44 for Sb(III) (300–100 μM), As(III) (14–10 mM), Cd(II) (2–0.9 mM), and Cu(II) (4–3 mM). The MICs for Sb(III), As(III), and Cu(II) of the strain iscR-280C could be restored almost completely (Table S2). As for bacterial H_2_O_2_ resistance, iscR-280 was less resistant than strains S44 and iscR-280C (Figure S4).

### IscR interacted with *iscRSUA* promoter region

**Figure 3:**
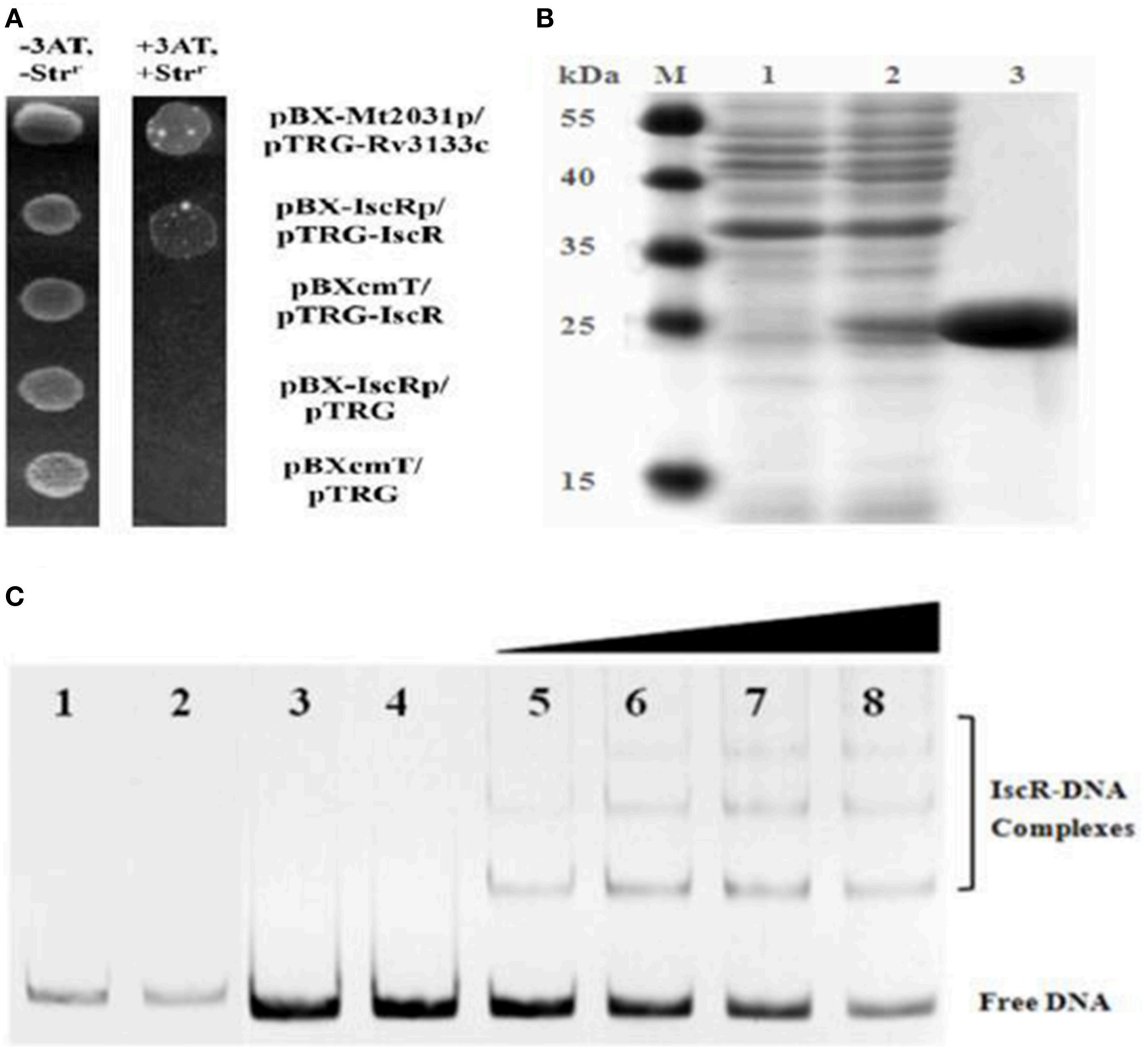
**B1H system and EMSA analysis for IscR interaction with DNA**. **(A)** B1H system for *iscR*. Co-transformants containing pBX-Mt2031p/pTRG-Rv3133c were employed as positive controls (CK+), while co-transformants containing pBXcmT/pTRG-IscR, pBX-*iscR*p/pTRG, and pBXcmT/pTRG were used as negative controls (CK-). Cells of CK+, pBX-*iscR*p/pTRG-IscR, and CK- were grown to OD_600_ of ~1.0 and 4 μL of each was spotted onto His-selective medium (+3AT, +Str^*r*^) and LB plate (-3AT, -Str^*r*^). **(B)** SDS-PAGE gel of purified IscR. Bands 1–3 show un-induced protein, IPTG-induced protein and the purified IscR, respectively. The protein size marker (kDa, Thermo Scientific) is shown on the left. **(C)** EMSA analysis of IscR interaction with DNA. Band 1–4 represent negative controls, 1, DNA probe containing no IscR binding motif (non-specific DNA probe) added with IscR; 2, non-specific DNA probe only; 3, DNA probe containing putative IscR binding motif (specific DNA probe) added with heat-inactivated IscR; 4, specific DNA probe only; Band 5–8 represent the 0.8, 1.6, 3.2, and 6.4 μg of IscR added with specific DNA probe, respectively.

The predicted promoter region sufficient for IscR binding was located within −181 to −117 nt upstream from the predicted *iscR* start codon. The B1H system assay revealed the interaction between IscR and its promoter region (Figure 3A). Each co-transformant with pBX-Mt2031p/pTRG-Rv3133c and pBX-*iscR*p/pTRG-IscR grew well in the screening medium. By contrast, no growth was observed for the negative controls. All of the co-transformants showed a normal growth trend on LB plates.

**Figure 4:**
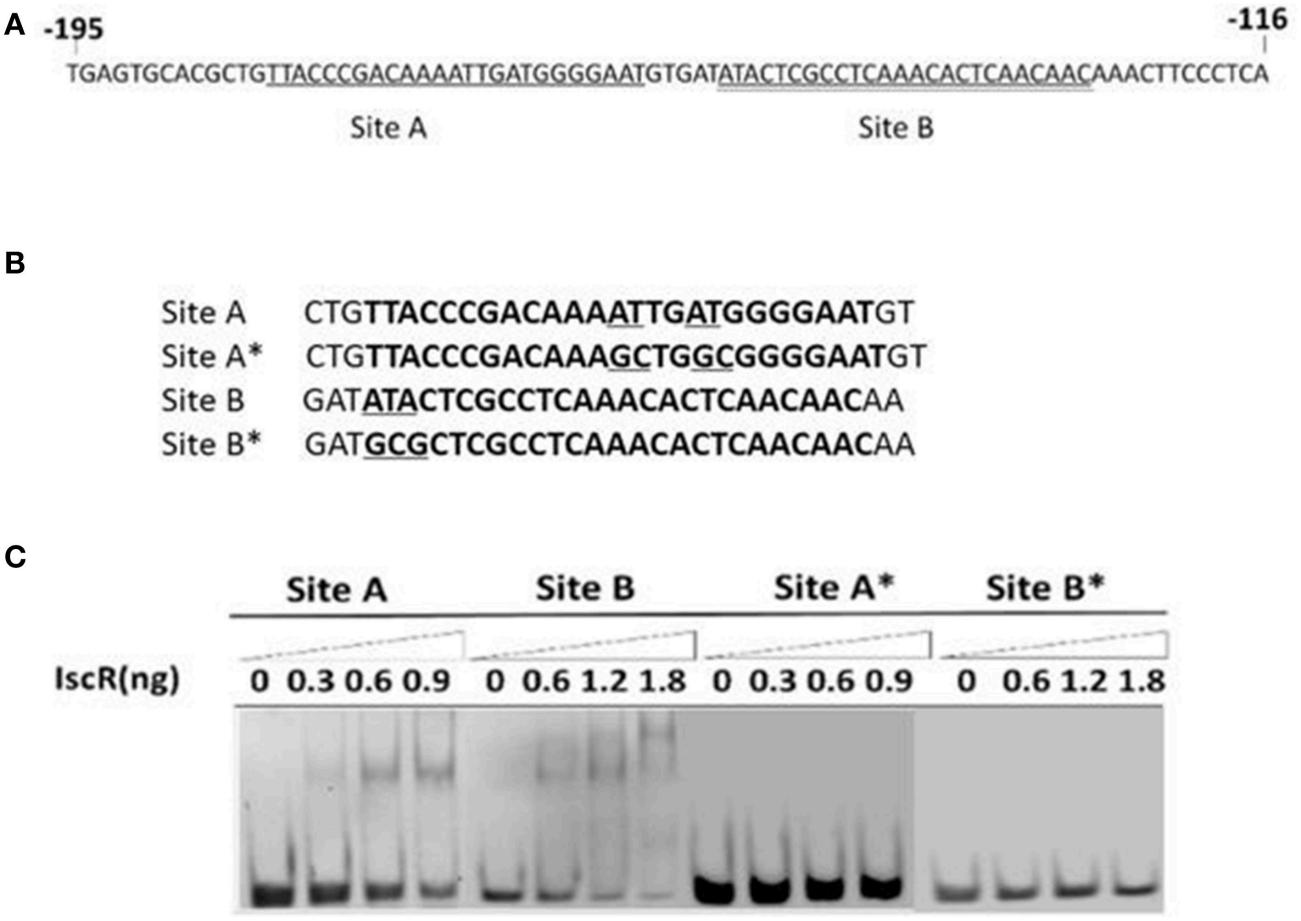
**EMSA for DNA-binding activity of IscR based on site-specific mutagenesis**. The IscR binding sites A (single underlined) and B (double underlined) were marked **(A)**. Four DNA substrates with or without conserved bases were synthesized and the mutated bases were underlined **(B)**. IscR is capable of binding with DNA substrates containing Site A or Site B motif, but each mutational motif of Site A* and Site B* lost binding ability **(C)**.

EMSA coupled with a purified recombinant IscR (Figure 3B) and a predicted regulatory sequence confirmed that IscR was able to bind the promoter region of the *isc* operon (Figure 3, Band 5–8). But negative controls did not show any lagging bands (Figure 3, Band 1–4). Comparative analysis using the RegPrecise database revealed two putative type I IscR-binding motifs in *C. testosteroni* S44 (Table S2, Figure 4A), denoted as site A (TTACCCGACAAAATTGATGGGGAAT, −182 to −158 bp relative to *iscR* start codon) and site B (ATACTCGCCTCAAACACTCAACAAC, −152 to −128 bp relative to *iscR* start codon), which was consistent with the predicted region in BDGP. Sequence blast of site A and site B against *C. testosteroni* S44 genome revealed some sequence hits (12–13 bp long) distributed before or within multiple genes (Table S3). Table S4 showed that the nucleotide sequences of IscR-binding motifs varied among different species (data extracted from the RegPrecise database, Novichkov et al., 2010).

In order to determine the precise binding sequence of IscR on its target genes, two 30-bp-sequences (Site A and Site B) of IscR-binding motifs (Figure 4B), were synthesized to proceed with EMSA. As expected, both of them could bind IscR (Figure 4C). Site-directed mutations were constructed to determine their effect on DNA-IscR interaction. Site B is representative of the Type I IscR binding site. No binding activity was detected after the highly conserved bases of ATA within the site B were substituted with GCG (Figure 4B). Site A is not as well-conserved and does not resemble other known IscR binding sites. However, mutations to the base pairs located at positions −169 (T), −170 (A), −165 (T), and −166 (A) within site A, eliminated IscR binding (Figure 4C).

### Sb(III) induced transcription of *iscRS*

**Figure 5:**
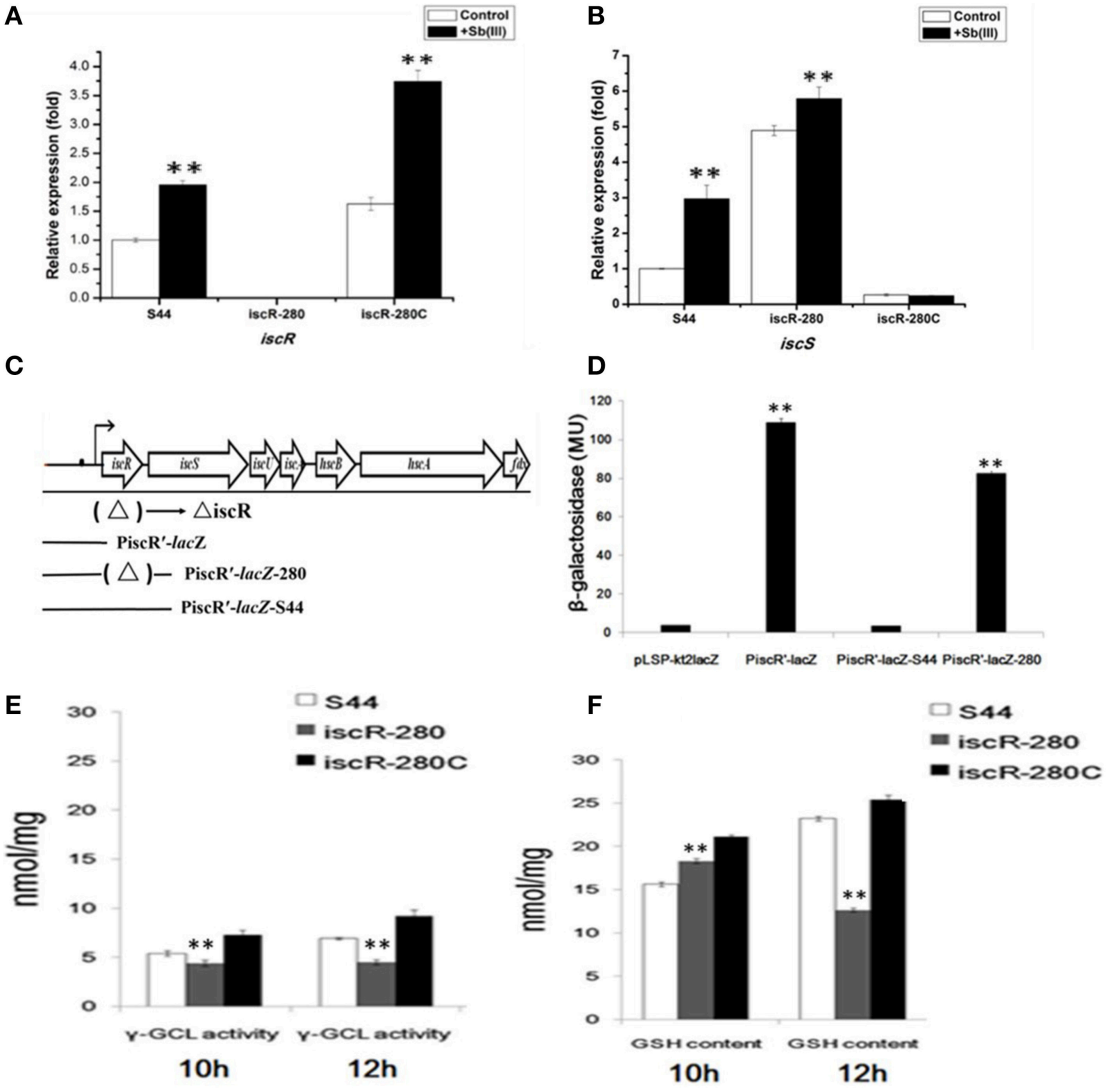
**Analyses of real-time RT-PCR and *Lac*Z reporter fusions for *iscRS* and γ-GCL activity/GSH content assay**. **(A,B)** represent real-time RT-PCR results for *iscR* and *iscS*, respectively. The relative mRNA expression levels of *iscR* and *iscS* induced by Sb(III) (black bars) and without induction (white bars) were determined as described in Materials and Methods. **(C)** The location of the DNA segments used to generate *lacZ* fusions. **(D)** β-galactosidase activity was measured from *E. coil* DH5α containing different *lacZ* fusions. *Lac*Z reporter fusions were constructed by ligating pLSP-kt2*LacZ* with *iscR* promoter (PiscR′-*lac*Z); with *iscR* promoter and *iscR* coding region of strain S44 (PiscR′-*lacZ*-S44); and with *iscR* promoter and *iscR* coding region of strain iscR-280 (PiscR′-*lacZ*-280). The empty vector pLSP-kt2*LacZ* was used as a control. **(E,F)** represent the determination of γ-GCL activity and GSH content, respectively. γ-GCL activity and GSH concentrations were calculated against a standard curve of GSH and expressed as the GSH concentration (nmol GSH/mg protein) obtained from cell free extract of the exponentially growing cells (mid-log phage at 10 h and stationary phase at 12 h). Data are expressed as mean ± SD, *N* = 3. Error bars represent standard deviations of triplicate tests. ^**^Indicates a significant difference from the control (*p* < 0.01, Student's *t*-test).

Real-time RT-PCR analyses revealed that the transcription of *iscR* and *iscS* in strain S44 was significantly promoted by Sb(III) (Figures 5A,B), respectively. As expected, *iscR* transcription was not detected in iscR-280 (Figure 5A). Moreover, *iscR* transcription in iscR-280C was also induced by Sb(III). Under non-inducing conditions, the transcription level of *iscS* in iscR-280 was 3.8-fold higher than in strain S44 (Figure 5B). Sb(III) slightly enhanced the *iscS* expression in strain iscR-280 (Figure 5B). The multicopy-based complementation of *iscR* in strain iscR-280C showed strong repression of *iscS* transcription (Figure 5B).

### IscR negatively regulated *iscRSUA* expression

The β-galactosidase activities were measured in planktonic cultures of *E. coli* DH5α containing the different *lac*Z fusions (Figures 5C,D). Figure 5D showed background β-galactosidase activity in pLSP-kt2*lacZ* (empty vector control) and PiscR′-*lacZ*-S44 (vector harboring intact *iscR* and *iscR* promoter), indicating that IscR negatively regulated transcription of *iscRSUA*. The *lac*Z fusions of both PiscR′-*lacZ* (vector harboring only the *iscR* promoter) and PiscR′-*lacZ*-280 (vector harboring Tn5-insertional mutant of *iscR* and the *iscR* promoter) exhibited high β-galactosidase activity (Figure 5D).

### γ-GCL activity and GSH content were both decreased in the *iscR* mutant

In order to determine whether thiol metabolism is related to the stress caused by Sb(III), we determined both the γ-GCL activity and GSH content simultaneously. The results demonstrated that the γ-GCL activity of iscR-280 was much lower than that of S44 and iscR-280C (Figure 5E), and the GSH content was positively correlated with γ-GCL activity (Figure 5F). The γ-GCL activities of iscR-280 at the two time points (10 h and 12 h) were nearly the same (Figures 5E,F), while the GSH content in iscR-280 was obviously reduced after being incubated for two more hours which might partially correspond to a point where there is an increased demand for GSH. The results indicated the *iscR* mutation led to a decreasing γ-GCL activity and less GSH content. Thiol metabolism might be influenced by the Isc system.

## Discussion

*C. testosteroni* S44 displayed resistance to multiple metal(loid)s, such as Sb(III), As(III), Cd(II), and Cu(II) (Xiong et al., 2011) and was also able to oxidize Sb(III). Whole genome sequencing of strain S44 revealed three As(III)/Sb(III) resistance genes including two putative *arsB* and one *glpF*. Although As and Sb share similar physicochemical properties, strain S44 displayed no As(III) oxidation. This is different as in the case of *A*. *tumefaciens* 5A, in which both As(III) and Sb(III) oxidation were found to occur in the same strain (Lehr et al., 2007). Moreover, the whole genome of strain S44 did not contain genes encoding the putative As(III) oxidase gene *aioBA* (Wang et al., 2015), which indicated that there were different mechanisms for bacterial oxidation of Sb(III) or As(III).

We observed that the disruption of *iscR* resulted in a lower level of resistance to Sb(III), As(III), Cd(II), Cu(II), and H_2_O_2_ and a reduced Sb(III) oxidation rate. This indicated that IscR is an important component of resistance to multiple metal(loid)s and also Sb(III) oxidation. Our finding also suggested *C. testosteroni* IscR plays an important role in the H_2_O_2_-induced oxidative stress response, similar as reported in *P. aeruginosa* and *V. vulnificus* (Lim and Choi, 2014; Romsang et al., 2014). Heavy-metals such as Cd(II) and Cu(II) could cause considerable oxidative stress by damaging Fe-S cluster assembly thereby generating free Fe(II) (Chillappagari et al., 2010; Xu and Imlay, 2012; Zheng et al., 2014). Sb(III) and As(III) might also lead to the destruction of Fe-S clusters. This again would increase unincorporated iron leading to substantially accelerated hydroxyl radical (OH·) formation by the Fenton reaction (Kohanski et al., 2007).

Our results showed that IscR could positively contribute to γ-GCL activity and GSH formation, possibly through regulating IscS-mediated cysteine desulfurization (Giel et al., 2006). Recent studies discovered that GSH alone can coordinate and stabilize Fe-S cluster formation under physiological conditions, and maturation of cytosolic Fe-S proteins required GSH (Qi et al., 2012; Wang et al., 2012). The Fe-S assembly protein IscU catalyzed formation of [2Fe-2S](GS)_4_ from Fe and S ions in the presence of GSH (Qi et al., 2012). Thus, in strain iscR-280, the inactivation of IscR resulted in up-regulation of the *isc* operon consuming more GSH during a specific period and eliminating more S from cysteine. Moreover, the GSH synthesis was partially blocked because of cysteine shortage. Thiol metabolisms protect microbe from oxidative damage caused by due oxidants such as heavy metals (Rouhier et al., 2008). Glutathione is one of the most abundant thiolates in proteobacteria and cyanobacteria serving as a protecting agent (Fahey and Sundquist, 1991). Antimonites [Sb(III) compounds], cadmic compound [Cd(II) compounds], and cupric compounds [Cu(II) compounds] were able to inactivate [Fe-S] enzymes via a oxidative-stress-dependent disabling of their [Fe–S] catalytic clusters (Calderon et al., 2009; Chillappagari et al., 2010; Xu and Imlay, 2012). A reducing environment and the presence of GSH within the cells was assumed to be a vital component of the bacterial oxidative stress response, indicating that IscR played global roles in resistances to multi-metal(loid)s. In addition, the decrease of Sb(III) oxidation rate and *in vitro* Sb(III) oxidation suggested that Sb(III) was potentially oxidized to less toxic Sb(V) as a detoxification mechanism by soluble extracellular enzyme.

IscR was reported to suppress the transcription initiation of the *isc* operon, including *iscR* (Schwartz et al., 2001). In this study, B1H system and EMSA analysis implied the auto-regulation of *iscR* expression indicating that IscR auto-regulated the Isc pathway for biosynthesis of its cofactor [Fe–S], which developed a connection between IscR activity and Fe–S clusters demand. Besides, we further confirmed that two putative IscR-binding motifs are essential for the recognition of IscR. Real-time RT-PCR analysis indicated the fact IscR may function as both transcriptional repressor and activator depending on cellular redox state. Under non-inducing conditions, IscR may function as a transcriptional repressor to maintain Fe-S cluster homeostasis. Under Sb(III) stress, *iscRS* expression in strain S44 was significantly promoted as IscR may function as a transcriptional activator. It has been reported that ROS can induce *iscR* expression (Imlay, 2006). The Fe-S cluster assembly system of Isc (*iscRSUA-hscBA-fdx*) was also up-regulated in the bacterial transcriptional response to oxidative stress (Wang et al., 2009). These indicated that intracellular oxidative stress originating from cellular exposure to Sb(III) might target Fe-S clusters and disable the [Fe-S] assembly. Meanwhile, under oxidative stress, most IscRs existed as apo-IscRs and functioned as an activator (Yeo et al., 2006), which in turn led to an increased expression of the Isc system to meet the demands for Fe–S biogenesis and for the acclimation to Sb(III)-induced toxicity.

**Figure 6:**
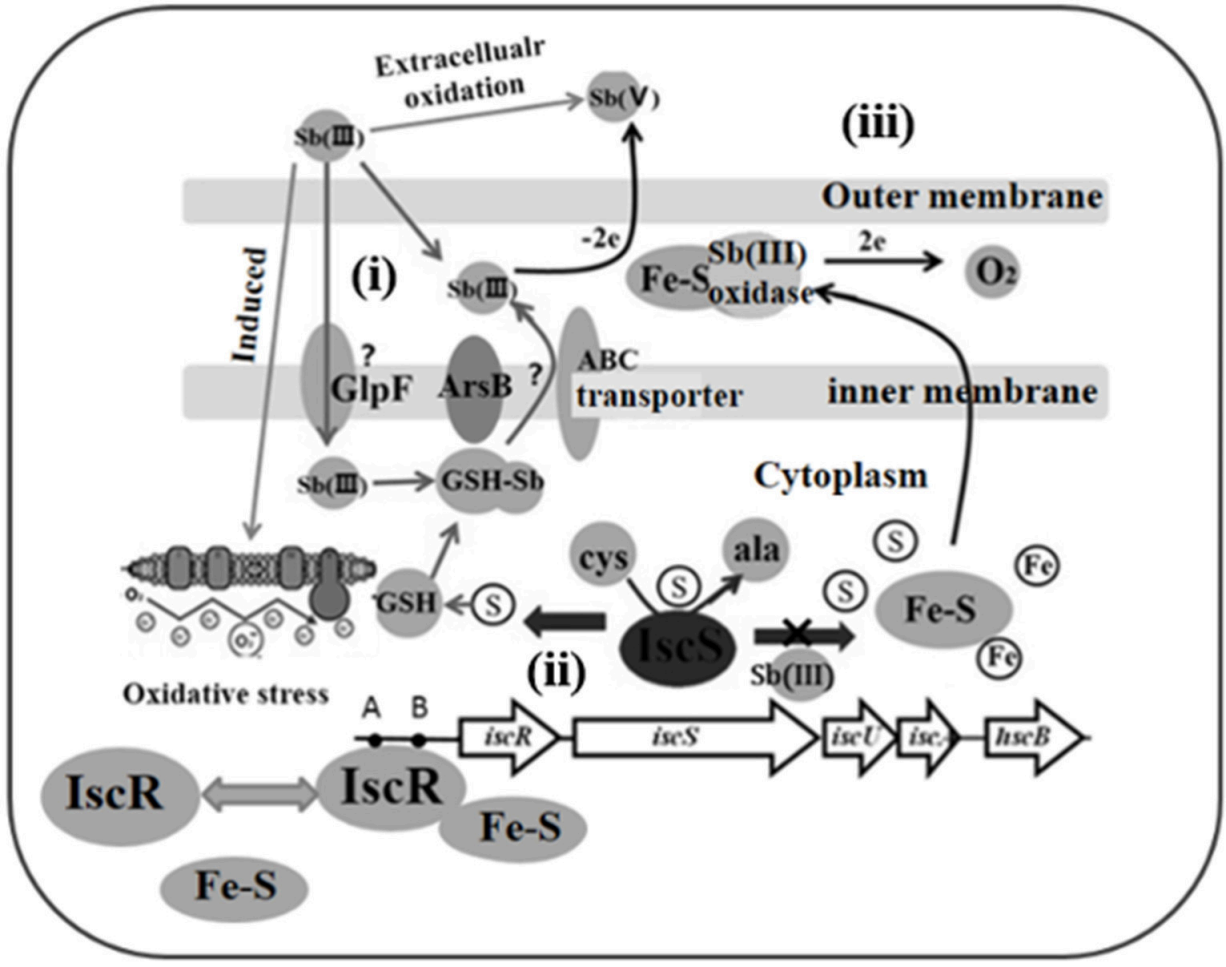
**A hypothetical model of IscR role in Sb(III) resistance and oxidation**. (i) Transport, Sb(III) is taken up by GlpF/ABC-like transporter and pumped out by ArsB (Meng et al., 2004); (ii) Toxicity, Sb(III)-caused toxicity disturbs the cellular redox homeostasis and damages the Fe-S cluster biogenesis. Meanwhile, impairment of IscR and elevated expression of IscS will result in a decrease of γ-GCL activity and GSH content; (iii) Restoration, intact IscR positively regulates the γ-GCL activity and GSH biosynthesis, and in turn the GSH promotes Fe-S cluster assembly and reduces the toxicity of Sb(III). Expelling Sb(III) is oxidized to less toxic Sb(V) by Fe-S containing enzymes.

In summary, based on the literatures and our data, we show a hypothetical model of IscR role in Sb(III) resistance and oxidation (Figure 6): (i) Sb(III) is taken by GlpF-like transporter, and secreted by ArsB in bacteria (Meng et al., 2004); (ii) Sb(III) induced toxicity disturb the cellular redox homeostasis and damage the Fe-S cluster biogenesis. Destruction of Fe-S clusters increases free iron enough to accelerate Fenton reaction to produce more toxic OH· (Dwyer et al., 2009). Therefore, a number of Fe-S proteins or enzymes, vital for cell growth and metabolism, are impaired or inactivated. Meanwhile, impairment of IscR and elevated expression of IscS result in a decrease of γ-GCL activity and GSH content. (iii) The complemented IscR positively regulates the γ-GCL activity and GSH biosynthesis, and in turn GSH promotes the Fe-S cluster assembly and stability. Sb(III) is finally oxidized to less toxic Sb(V) potentially by extracellularly secreted proteins employing Fe-S cluster as cofactors. Consequently, Fe-S cluster synthesis, holo-IscR assembling and Fe-S protein function are progressively restored, which leads to the recovery of cellular redox homeostasis.

## Author contributions

HL and WZ designed and performed the experiments and wrote the manuscript. SZ, JH, and JL participated in the experiments. CR helped to design the study and revise the manuscript. GW design the study and revised the manuscript. All authors read and approved the final manuscript.

### Conflict of interest statement

The authors declare that the research was conducted in the absence of any commercial or financial relationships that could be construed as a potential conflict of interest.

## References

[B1] Calderon I. L. Elías A. O. Fuentes E. L. Pradenas G. A. Castro M. E. Arenas F. A.. (2009). Tellurite-mediated disabling of [4Fe-4S] clusters of *Escherichia coli* dehydratases. Microbiology 155, 1840–1846. 10.1099/mic.0.026260-0 19383690

[B2] Chillappagari S. A. Trip H. Kuipers O. P. Marahiel M. A. Miethke M. (2010). Copper stress affects iron homeostasis by destabilizing iron-sulfur cluster formation in *Bacillus subtilis*. J. Bacteriol. 192, 2512–2524. 10.1128/JB.00058-10 20233928 PMC2863568

[B3] Decuypere S. Vanaerschot M. Brunker K. Imamura H. Muler S. Khanal B.. (2012). Molecular mechanisms of drug resistance in natural *Leishmania* populations vary with genetic background. PLoS Negl. Trop. Dis. 6:e1514. 10.1371/journal.pntd.0001514 22389733 PMC3289598

[B4] Dwyer D. J. Kohanski M. A. Collins J. J. (2009). Role of reactive oxygen species in antibiotic action and resistance. Curr. Opin. Microbiol. 12, 482–489. 10.1016/j.mib.2009.06.018 19647477 PMC2761529

[B5] Fahey R. C. Sundquist A. R. (1991). Evolution of glutathione metabolism, in Advances in Enzymology and Related Areas of Molecular Biology, Vol. 64, ed Meister A. (Hoboken, NJ: John Wiley & Sons, Inc.), 1–53. 10.1002/9780470123102.ch1 1675828

[B6] Filella M. Belzile N. Chen Y. W. (2002). Antimony in the environment: a review focused on natural waters I. Occurrence. Earth Sci. Rev. 57, 125–176. 10.1016/S0012-8252(01)00070-8

[B7] Filella M. Belzile N. Lett M. C. (2007). Antimony in the environment, A review focused on natural waters. III. Microbiota relevant interactions. Earth Sci. Rev. 80, 195–217. 10.1016/j.earscirev.2006.09.003

[B8] Fleischhacker A. S. Stubna A. Hsueh K. L. Guo Y. Teter S. J. Rose J. C.. (2012). Characterization of the [2Fe-2S] cluster of *Escherichia coli* transcription factor IscR. Biochemistry 51, 4453–4462. 10.1021/bi3003204 22583201 PMC3447993

[B9] Gao C. Yang M. He G. (2012). Characterization of a novel ArsR-like regulator encoded by Rv2034 in *Mycobacterium tuberculosis*. PLoS ONE 7:e36255. 10.1371/journal.pone.0036255 22558408 PMC3338718

[B10] Giel J. L. Nesbit A. D. Mettert E. L. Fleischhacker A. S. Wanta B. T. Kiley P. J. (2013). Regulation of iron-sulphur cluster homeostasis through transcriptional control of the Isc pathway by [2Fe-2S]-IscR in *Escherichia coli*. Mol. Microbiol. 87, 478–492. 10.1111/mmi.12052 23075318 PMC4108476

[B11] Giel J. L. Rodionov D. Liu M. Blattner F. R. Kiley P. J. (2006). IscR-dependent gene expression links iron-sulphur cluster assembly to the control of O_2_-regulated genes in *Escherichia coli*. Mol. Microbiol. 60, 1058–1075. 10.1111/j.1365-2958.2006.05160.x 16677314

[B12] Guo M. Feng H. Zhang J. Wang W. Wang Y. Li Y.. (2009). Dissecting transcription regulatory pathways through a new bacterial one-hybrid reporter system. Genome Res. 19, 1301–1308. 10.1101/gr.086595.108 19228590 PMC2704442

[B13] Herbst K. A. Rose G. Hanusch K. Schumann H. Wolf H. U. (1985). Antimony and antimony compounds, in The Ullmann's Encyclopedia of Industrial Chemistry, eds Elvers B. Hawkins S. Russey W. (Weinheim: VCH Publishers), 55–76.

[B14] Huang H. C. He S. Y. Bauer D. W. Collmer A. (1992). The *Pseudomonas syringae* pv. syringae 61 *hrpH* product, an envelope protein required for elicitation of the hypersensitive response in plants. J. Bacteriol. 174, 6878–6885. 1400238 10.1128/jb.174.21.6878-6885.1992 PMC207366

[B15] Imlay J. A. (2006). Iron-sulphur clusters and the problem with oxygen. Mol. Microbiol. 59, 1073–1082. 10.1111/j.1365-2958.2006.05028.x 16430685

[B16] Kang Y. S. Heinemann J. Bothner B. Rensing C. McDermott T. R. (2012). Integrated co-regulation of bacterial arsenic and phosphorus metabolisms. Environ. Microbiol. 14, 3097–3109. 10.1111/j.1462-2920.2012.02881.x 23057575

[B17] Kohanski M. A. Dwyer D. J. Hayete B. Lawrence C. A. Collins J. J. (2007). A common mechanism of cellular death induced by bactericidal antibiotics. Cell 130, 797–810. 10.1016/j.cell.2007.06.049 17803904

[B18] Larsen R. A. Wilson M. M. Guss A. M. Metcalf W. W. (2002). Genetic analysis of pigment biosynthesis in *Xanthobacter autotrophicus* Py2 using a new, highly efficient transposon mutagenesis system that is functional in a wide variety of bacteria. Arch. Microbiol. 178, 193–483. 10.1007/s00203-002-0442-2 12189420

[B19] Legare D. Richard D. Mukhopadhyay R. Stierhof Y. D. Rosen B. P. Haimeur A.. (2001). The *Leishmania* ATP binding cassette protein PGPA is an intracellular metal-thiol transporter ATPase. J. Biol. Chem. 276, 26301–26307. 10.1074/jbc.M102351200 11306588

[B20] Lehr C. R. Kashyap D. R. McDermott T. R. (2007). New insights into microbial oxidation of antimony and arsenic. Appl. Environ. Microbiol. 73, 2386–2389. 10.1128/AEM.02789-06 17308197 PMC1855643

[B21] Li J. Wang Q. Li M. Yang B. Shi M. Guo W.. (2015). Proteomics and genetics for identification of a bacterial antimonite oxidase in *Agrobacterium tumefaciens*. Environ. Sci. Technol. 49, 5980–5989. 10.1021/es506318b 25909855

[B22] Li J. Wang Q. Zhang S. Qin D. Wang G. (2013). Phylogenetic and genome analyses of antimony-oxidizing bacteria isolated from antimony mined soil. Int. Biodeter. Biodegr. 76, 76–80. 10.1016/j.ibiod.2012.06.009

[B23] Lialikova N. N. (1974). *Stibiobacter senarmontii*–a new microorganism oxidizing antimony. Mikrobiologiia 43, 941–943. 4449497

[B24] Lim J. G. Choi S. H. (2014). IscR is a global regulator essential for the pathogenesis of *Vibrio vulnificus* and induced by host cells. Infect. Immun. 82, 569–578. 10.1128/IAI.01141-13 24478072 PMC3911388

[B25] Liu H. L. Huang J. Zhang S. Z. Xu B. Wang G. J. (2015). Chromate interaction with the chromate reducing actinobacterium *Intrasporangium chromatireducens* Q5-1. *Geomicrobiol*. J. 32, 616–623. 10.1080/01490451.2014.971200

[B26] Meng Y. L. Liu Z. Rosen B. P. (2004). As(III) and Sb(III) uptake by GlpF and efflux by ArsB in *Escherichia coli*. J. Biol. Chem. 279, 18334–18341. 10.1074/jbc.M400037200 14970228

[B27] Miller J. H. (1972). Experiments in Molecular Genetics. New York, NY: Cold Spring Harbor Laboratory Press.

[B28] Miller V. L. Mekalanos J. J. (1988). A novel suicide vector and its use in construction of insertion mutations, osmoregulation of outer membrane proteins and virulence determinants in *Vibrio cholera* requires *toxR*. J. Bacteriol. 170, 2575–2583. 2836362 10.1128/jb.170.6.2575-2583.1988 PMC211174

[B29] Novichkov P. S. Laikova O. N. Novichkova E. S. Gelfand M. S. Arkin A. P. Dubchak I.. (2010). RegPrecise, a database of curated genomic inferences of transcriptional regulatory interactions in prokaryotes. Nucleic Acids Res. 38, D111–D118. 10.1093/nar/gkp894 19884135 PMC2808921

[B30] Pfaffl M. W. (2001). A new mathematical model for relative quantification in real-time RT-PCR. Nucleic Acids Res. 29, 2002–2007. 10.1093/nar/29.9.e45 PMC55695 11328886

[B31] Qi W. Li J. Chain C. Y. Pasquevich G. A. Pasquevich A. F. Cowan J. A. (2012). Glutathione complexed Fe-S centers. J. Am. Chem. Soc. 134, 10745–10748. 10.1021/ja302186j 22687047 PMC3401418

[B32] Rajagopalan S. Teter S. J. Zwart P. H. Brennan R. G. Phillips K. J. Kiley P. J. (2013). Studies of IscR reveal a unique mechanism for metal-dependent regulation of DNA binding specificity. Nat. Struct. Mol. Biol. 20, 740–747. 10.1038/nsmb.2568 23644595 PMC3676455

[B33] Reese M. G. (2001). Application of a time-delay neural network to promoter annotation in the *Drosophila melanogaster* genome. Comput. Chem. 26, 51–56. 10.1016/S0097-8485(01)00099-7 11765852

[B34] Rincon-Enriquez G. Crété P. Barras F. Py B. (2008). Biogenesis of Fe/S proteins and pathogenicity: IscR plays a key role in allowing *Erwinia chrysanthemi* to adapt to hostile conditions. Mol. Microbiol. 67, 1257–1273. 10.1111/j.1365-2958.2008.06118.x 18284573

[B35] Romsang A. Duang-Nkern J. Leesukon P. Saninjuk K. Vattanaviboon P. Mongkolsuk S. (2014). The Iron-Sulphur cluster biosynthesis regulator IscR contributes to iron homeostasis and resistance to oxidants in *Pseudomonas aeruginosa*. PLoS ONE 9:e86763. 10.1371/journal.pone.0086763 24466226 PMC3899308

[B36] Rouhier N. Lemaire S. D. Jacquot J. P. (2008). The role of glutathione in photosynthetic organisms, emerging functions for glutaredoxins and glutathionylation. Annu. Rev. Plant Biol. 59, 143–166. 10.1146/annurev.arplant.59.032607.092811 18444899

[B37] Schwartz C. J. Giel J. L. Patschkowski T. Luther C. Ruzicka F. J. Beinert H.. (2001). IscR, a Fe-S cluster-containing transcription factor, represses expression of *Escherichia coli* genes encoding Fe-S cluster assembly proteins. Proc. Natl. Acad. Sci. U.S.A. 98, 14895–14900. 10.1073/pnas.251550898 11742080 PMC64955

[B38] Shotyk W. Krachler M. Chen B. Zheng J. (2005). Natural abundance of Sb and Se in pristine groundwaters, Springwater Township, Ontario, Canada, and implications for tracing contamination from landfill leachates. J. Environ. Monit. 7, 1238–1244. 10.1039/b509352j 16307077

[B39] Simon R. Priefer U. Pühler A. (1983). A broad host range mobilization system for *in vivo* genetic engineering transposon mutagenesis in gram negative bacteria. Nat. Biotechnol. 1, 784–791. 10.1038/nbt1183-784

[B40] Thompson J. D. Gibson T. J. Plewniak F. Jeanmougin F. Higgins D. G. (1997). The CLUSTAL_X windows interface, flexible strategies for multiple sequence alignment aided by quality analysis tools. Nucleic Acids Res. 25, 4876–4882. 10.1093/nar/25.24.4876 9396791 PMC147148

[B41] Tokumoto U. Takahashi Y. (2001). Genetic analysis of the *isc* operon in *Escherichia coli* involved in the biogenesis of cellular iron-sulfur proteins. J. Biochem. 130, 63–71. 10.1093/oxfordjournals.jbchem.a002963 11432781

[B42] Torma A. E. Gabra G. G. (1977). Oxidation of stibnite by *Thiobacillus ferrooxidans*. Antonie. Leeuw. Int. J. G. 43, 1–6. 10.1007/BF02316204 17364

[B43] Wang L. Ouyang B. Li Y. Feng Y. Jacquot J. P. Rouhier N.. (2012). Glutathione regulates the transfer of iron-sulfur cluster from monothiol and dithiol glutaredoxins to apo ferredoxin. Protein Cell 3, 714–721. 10.1007/s13238-012-2051-4 22886498 PMC4875373

[B44] Wang Q. Warelow T. P. Kang Y.-S. Romano C. Osborne T. H. Lehr C. R.. (2015). Arsenite oxidase also functions as an antimonite oxidase. Appl. Environ. Microbiol. 81, 1959–1965. 10.1128/AEM.02981-14 25576601 PMC4345363

[B45] Wang S. Deng K. Zaremba S. Deng X. Lin C. Wang Q.. (2009). Transcriptomic response of *Escherichia coli* O157:H7 to oxidative stress. Appl. Environ. Microbiol. 75, 6110–6123. 10.1128/AEM.00914-09 19666735 PMC2753066

[B46] Weeger W. Lievremont D. Perret M. Lagarde F. Hubert J. C. Leroy M.. (1999). Oxidation of arsenite to arsenate by a bacterium isolated from an aquatic environment. Biometals 12, 141–149. 10.1023/A:1009255012328 10406083

[B47] White C. C. Viernes H. Krejsa C. M. Botta D. Kavanagh T. J. (2003). Fluorescence-based microtiter plate assay for glutamate-cysteine ligase activity. Anal. Biochem. 318, 175–180. 10.1016/S0003-2697(03)00143-X 12814619

[B48] Xiong J. Li D. Li H. He M. Miller S. Yu L.. (2011). Genome analysis and characterization of zinc efflux systems of a highly zinc resistant bacterium, *Comamonas testosteroni* S44. Res. Microbiol. 162, 671–679. 10.1016/j.resmic.2011.06.002 21704702

[B49] Xu F. F. Imlay J. A. (2012). Silver(I), mercury(II), cadmium(II), and zinc(II) target exposed enzymic iron-sulfur clusters when they toxify *Escherichia coli*. Appl. Environ. Microbiol. 78, 3614–3621. 10.1128/AEM.07368-11 22344668 PMC3346352

[B50] Yeo W. S. Lee J. H. Lee K. C. Roe J. H. (2006). IscR acts as an activator in response to oxidative stress for the *suf* operon encoding Fe-S assembly proteins. Mol. Microbiol. 61, 206–218. 10.1111/j.1365-2958.2006.05220.x 16824106

[B51] Zheng S. Su J. Wang L. Yao R. Wang D. Deng Y.. (2014). Selenite reduction by the obligate aerobic bacterium *Comamonas testosteroni* S44 isolated from a metal-contaminated soil. BMC Microbiol. 14:204. 10.1186/s12866-014-0204-8 25098921 PMC4236595

