# Supplementary material for: Global Regulator IscR Positively Contributes to Antimonite Resistance and Oxidation in Comamonas testosteroni S44
Source: Front Mol Biosci. 2015 Dec 18;2:70. doi: 10.3389/fmolb.2015.00070 (PMC4683182; doi:10.3389/fmolb.2015.00070)
Supplement: Supplementary file 1 [file DataSheet1.docx]

Supplementary Material

**Global regulator** **IscR positively contributes to** **antimonite resistance** **and oxidation in** ***Comamonas testosteroni* S44**

Hongliang Liu^1, 2#^, Weiping Zhuang^1#^, Shengzhe Zhang^1^, Christopher Rensing^3^, Jun Huang^1^, Jie Li^1^ and Gejiao Wang^1^*

* Correspondence: *Prof. Gejiao Wang*

E-mail: [gejiao@mail.hzau.edu.cn](mailto:gejiao@mail.hzau.edu.cn)

## Supplementary Figures


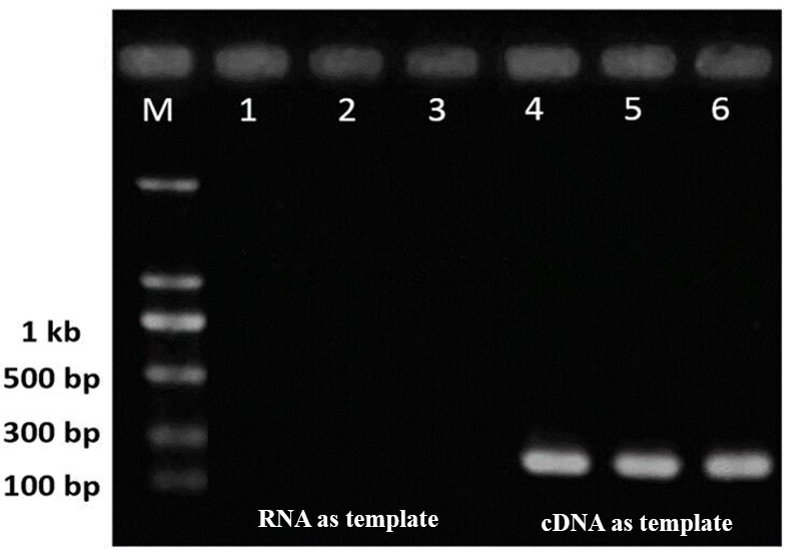


**Fig. S1.** Confirmation of no genomic DNA contaminating the total RNA. Lanes No. 1–6 display amplifications of 16S rRNA genes from S44 (1, 4), iscR-280 (2, 5) and iscR-280C (3, 6) using RNA (1–3) or cDNA (4–6) as template, respectively.


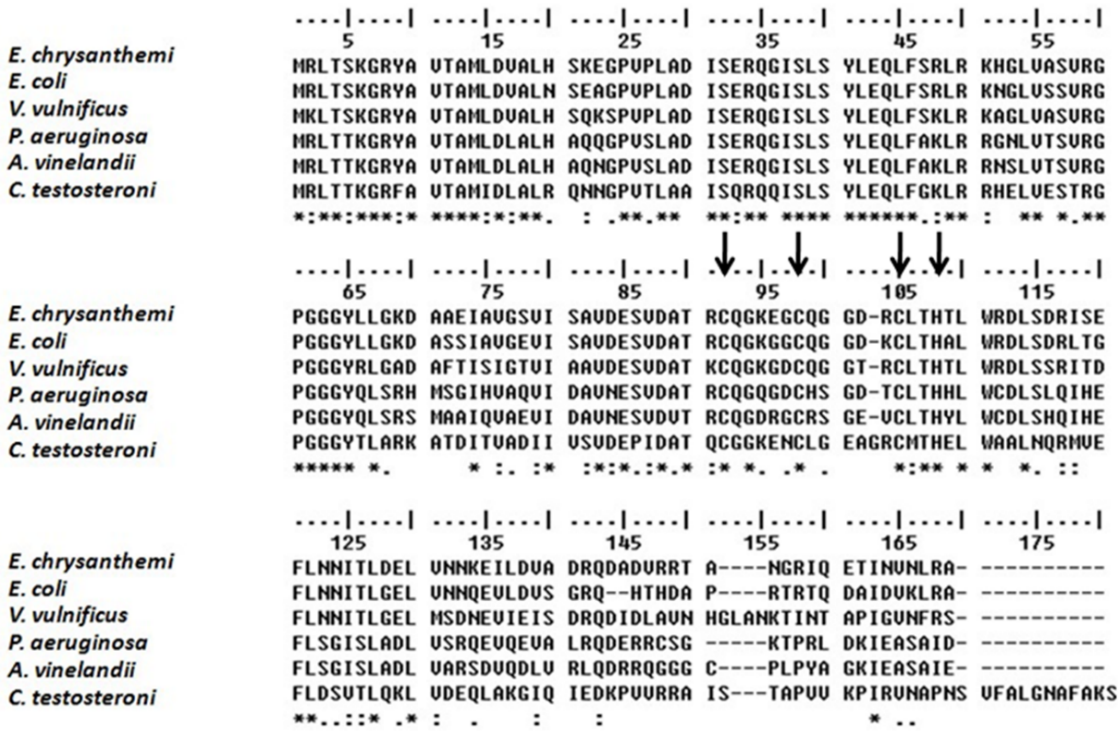


**Fig. S2.** **Schematic representation of multiple alignment of *C. testosteroni* IscR.** The alignment was performed using the Clustal_X algorithm. Black arrows indicate the conserved amino acids responsible for the Fe-S cluster ligation. The asterisks, colons, and periods represent identical residues, conserved substitutions, and semi-conserved substitutions, respectively. The number above the alignment indicates the position of the indicated amino acid.


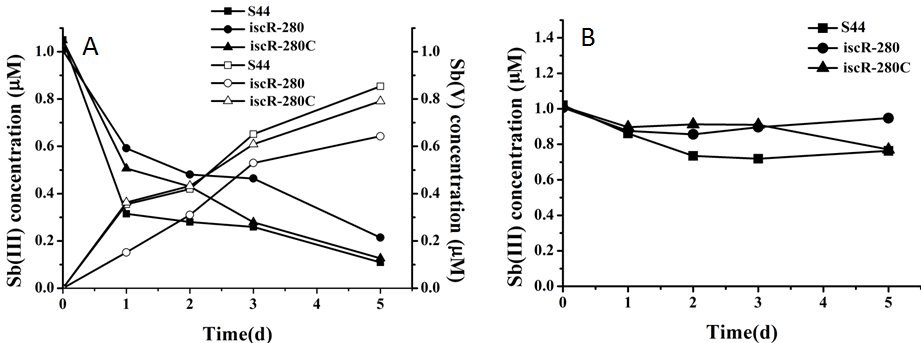


**Fig. S3. Sb(III) oxidation assay of culture supernatant and cell-free extract.** Two equal parts of culture supernatant (A) and cell-free extract (B) were supplemented with 1 μM Sb(III), and incubated for 5 d. Sb(III) (filled labels) and Sb(V) (hollow labels) concentrations were measured. Sb(III) oxidation occurred in the culture supernatant with oxidation rates of S44 > iscR-280C > iscR-280, while cell-free extract showed no obvious Sb(III) oxidation.


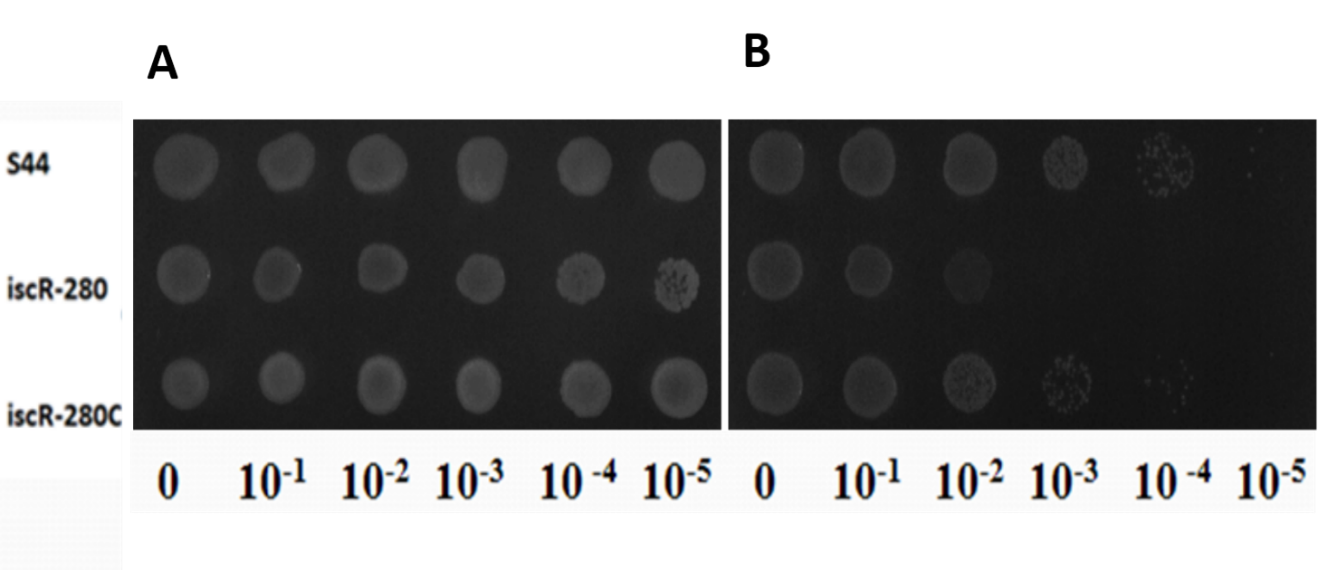


**Fig. S4.** Spotting assay to determine influence of H_2_O_2_ on growth of strains S44, iscR-280 and iscR-280C. Cells of strains S44, iscR-280 and iscR-280C were grown to OD_600_ of ~1.0 and 6 μL of each dilution was spotted onto LB plate without H_2_O_2_ (A) or supplemented with 0.3 mM H_2_O_2_ (B). 0-10^-5^ are different dilutions of the bacterial culture, respectively. The plates were aerobically incubated at 37 °C for 48 h.

## Supplementary Tables

**TABLE S1 List of primers used in this study**

| **Primer^a^** | **Primer sequence (5'-3')^b^** | **Usage** |
| --- | --- | --- |
| pRLS-F  pRLS-R  IscR-up-F | TGTAACGCACTGAGAAGC  GCGGGCAAGAATGTGAAT  AAATCTAGAAAGGGCGGAGACTGTGCT | Primer for inverse PCR  Primer for *iscR* complementary |
| IscR-down-R | AAAGGTACCATCGGAGTCGGGCTTCA |  |
| Cotrans-R-F | ATGCGTCTTACGACCAAAGG | A 348 bp of the *iscR* coding region |
| Cotrans-R-R | CTGATTCAGTGCAGCCCACA |  |
| Cotrans-S-F | GATCTGGAGCAGGTGTTCGT | A 253 bp of the *iscS* coding region |
| Cotrans-S-R | CAATTTCCTCTTCCGTCGTG |  |
| Cotrans-U-F | TCAAGGTCAATCCCGCCACT | A 204 bp of the *iscU* coding region |
| Cotrans-U-R | CTTCAGCCAGGATGGAGCAA |  |
| Cotrans-A-F | CGTCATGTGAACCGCTACCT | A 242 bp of the *iscA* coding region |
| Cotrans-A-R | TGGAACTTGAAGCCTTCGTT |  |
| Cotrans-RS-F | AAAAGCTGGTGGACGAGCAA | Sequence across *iscR*-*S* (270 bp) |
| Cotrans-RS-R | CCATCAGGGCGAAGAAACAT |  |
| Cotrans-SU-F | AGCCCTCTGTGGGAGATGTA | Sequence across *iscS*-*U* (253 bp) |
| Cotrans-SU-R | AGTGGCGGGATTGACCTTGA |  |
| Cotrans-UA-F | AAGGGCAAGACGCTGGATGA | Sequence across *iscU*-*A* (288 bp) |
| Cotrans-UA-R | TTGTAGGCCAAGCCGGAACA |  |
| IscR-pro-F | AAAGGATCCATGCGTCTTACGACCAAAGG | Primer for IscR expression in EMSA |
| IscR-pro-R | AAAAAGCTTTATCGGAGTCGGGCTTCA | and bacterial one-hybrid system |
| EMSA-F | ATCTCGGGGTTATTAGGTGA | Primer for *iscR* promoter fragment in EMSA |
| EMSA-R | CTTTGGTCGTAAGACGCATA | and bacterial one-hybrid system |
| RT-16SF | GTAGTCCACGCCCTAAACG | 16S rRNA gene of strain S44 for real-time RT-PCR analysis |
| RT-16SR | CCAACATCCACGACACGA |  |
| RT-iscRF | CAAGGAAAACTGTCTGGGCG | *iscR* for real-time RT-PCR analysis |
| RT-iscRR | TGCGTTCACACGGATGGG |  |
| RT-iscSF | CCCCGCACTTTCCCATTTAT | *iscS* for real-time RT-PCR analysis |
| RT-iscSR | GGCTTCTTCGGCTTCCCAG |  |
| PiscR-F  PiscR-R  S44PiscR+P-F  S44PiscR+P-R  iscR-280IscR+P-F  iscR-280IscR+P-R | AAA GGATCC GCACTACAACAGGCACG  AAA GGATCC TCCAGATAGGACAGCGAAA  AAA GGATCC CAGGCACTACAACAGGCACG  AAA GGATCC TTACCCAGGGCGAACACC  AAA GGATCC GGGGTTATTAGGTGAGTGC  AAA GGATCC CGAGATAAATGGGAAAGTGC | *iscR* promoter fragment for *LacZ* fusions assay  *iscR* promoter fragment with *iscR* gene of strain S44 for *LacZ* fusions assay  *iscR* promoter fragment with *iscR* of strain iscR-280 for *LacZ* fusions assay |

^a^ The primers were designed using the genomic sequence of the *C. testosteroni* S44 genome with the accession number of ADVQ00000000 (Xiong et al., 2011).

^b^ Regions of oligonucleotides not complementary to corresponding genes were underlined.

**TABLE S2 MICs of multi-metal(loid)s for strains S44, iscR-280 and iscR-280C.**

| **Strain^a^** | **Sb(III)** | **As(III)** | **Cd(II)** | **Cu(II)** |
| --- | --- | --- | --- | --- |
| S44 | 300 μM | 14 mM | 2 mM | 4 mM |
| iscR-280 | 100 μM | 10 mM | 0.9 mM | 3 mM |
| iscR-280C | 200 μM | 13 mM | 1 mM | 4 mM |

^a^ Strains S44, iscR-280 and iscR-280C were incubated in LB medium supplemented with an indicated concentration of metal(loid)s at 37 °C for 48 h.

**TABLE S3 Blast of putative IscR-binding motifs against** ***C. testosteroni* S44 genome**

| **Contig locus^a^** | **Matching sequence****^b^** | **Gene sequence location^c^** | |
| --- | --- | --- | --- |
| **Site A** |  | *iscR* promoter region | |
| Contig00061 | TTACCCGACAAAATTGATGGGGAAT |  |  |
| Contig00061 | CCGACAAAATTG | Methylmalonate-semialdehyde dehydrogenase  GntR family transcriptional regulator | |
| Contig00053 | CCCGACAAAATTG |  |  |
| Contig00105 | ACAAAATTGATG | NADH:flavin oxidoreductase/NADH oxidase  Sugar efflux transporter |  |
| Contig00069 | ATTGATGGGGAA |  |  |
| Contig00066 | ATTGATGGGGAA | Aldolase II superfamily protein  Hypothetical protein |  |
| Contig00039 | AAATTGATGGGG |  |  |
| **Site B** |  |  |  |
| Contig00061 | ATACTCGCCTCAAACACTCAACAAC | *iscR* promoter region |  |
| Contig00061 | TCAAACACTCAA | Phosphoglucose isomerase |  |
| Contig00046 | AACACTCAACAAC | Glutaredoxin-related protein  Hypothetical protein |  |
| Contig00102 | GCGTCAAACACT |  |  |
| Contig00088 | ACACTCAACAAC | Subtilisin-like serine proteases  50S ribosomal protein L20 |  |
| Contig00077 | CTCGCGTCAAAC |  |  |
| Contig00074 | TCAAACACTCAA | Diacylglycerol kinase  Gluconate-2-dehydrogenase |  |
| Contig00044 | CTCGCGTCAAAC |  |  |
| Contig00015 | TCAAACACTCAA | Periplasmic sensor hybrid histidine kinase |  |

^a^ Locus of putative IscR-binding motifs in *C. testosteroni* S44 genome.

^b^ Matching sequences were retrieved from *C. testosteroni* S44 genome.

^c^ Putative DNA binding motifs located before or within relative genes.

**TABLE S4 Putative IscR binding motifs in different species ^a^**

| **Species** | **IscR-binding motif 1** | **IscR-binding motif 2** |
| --- | --- | --- |
| *Comamonas testosteroni* | TTACCCGACAAAATTGATGGGGAAT | ATACTCGCCTCAAACACTCAACAAC  ATAGTTGACCTAATTACTCGGATAA |
| *Pseudomonas aeruginosa* | TTAGTTGACCATTTTTCTGGGACAA |  |
| *Escherichia coli* | ATACCCGACTAAATCAGTCAAGTAA | ATAGTTGACCAATTTACTCGGGAAT  ATACTTGACCATTTTGGTCAGGTAT |
| *Vibrio vulnificus* | ATACCTGACTATTTTAGTCAAATAA |  |
| *Erwinia chrysanthemi* | ATAGTTGAGTTATTTACTCGGTTAA | ATAGTTGACTGAAACACTCGGGAAT |
| *Azotobacter vinelandii* | ATAGTTGATCCTGTTTGTCGGGTAT |  |

^a^ Putative IscR binding motifs were retrieved from RegPrecise database (Novichkov et al., 2010) and performed blast using the NCBI BLAST server (<http://www.ncbi.nlm.nih.gov/>BLAST) against corresponding genomes of different species in GenBank.
